# Experienced mental workload, perception of usability, their interaction and impact on task performance

**DOI:** 10.1371/journal.pone.0199661

**Published:** 2018-08-01

**Authors:** Luca Longo

**Affiliations:** 1 School of Computing, College of health and sciences, Dublin Institute of Technology, Dublin, Ireland; 2 ADAPT: The global centre of excellence for digital content and media innovation, Dublin, Ireland; Universita degli Studi di Perugia, ITALY

## Abstract

Past research in HCI has generated a number of procedures for assessing the usability of interacting systems. In these procedures there is a tendency to omit characteristics of the users, aspects of the context and peculiarities of the tasks. Building a cohesive model that incorporates these features is not obvious. A construct greatly invoked in Human Factors is human Mental Workload. Its assessment is fundamental for predicting human performance. Despite the several uses of Usability and Mental Workload, not much has been done to explore their relationship. This empirical research focused on I) the investigation of such a relationship and II) the investigation of the impact of the two constructs on human performance. A user study was carried out with participants executing a set of information-seeking tasks over three popular web-sites. A deep correlation analysis of usability and mental workload, by task, by user and by classes of objective task performance was done (I). A number of Supervised Machine Learning techniques based upon different learning strategy were employed for building models aimed at predicting classes of task performance (II). Findings strongly suggests that usability and mental workload are two non overlapping constructs and they can be jointly employed to greatly improve the prediction of human performance.

## Introduction

**Fig 1:**
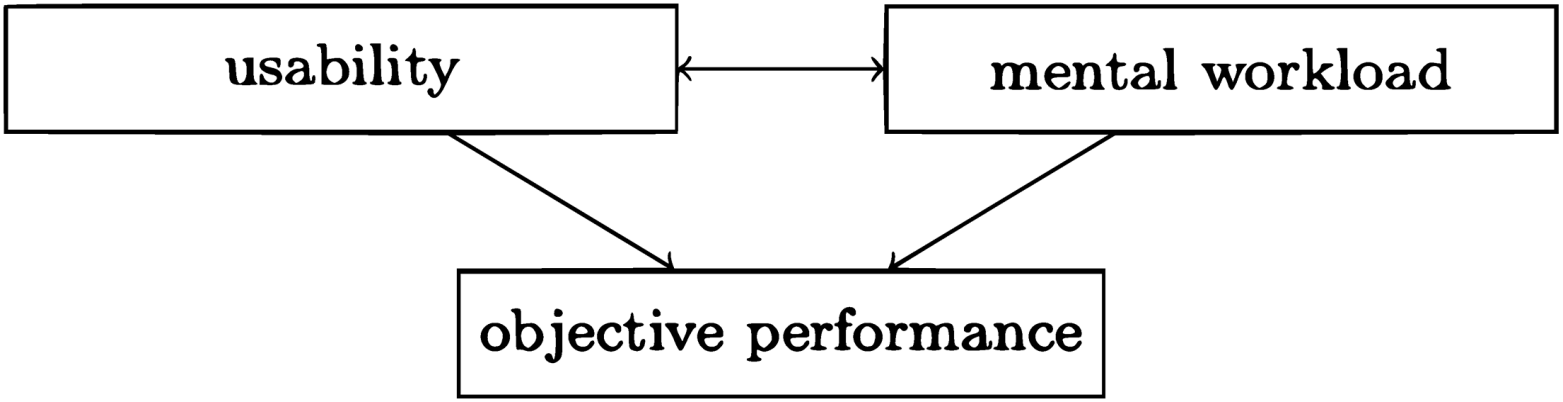
Schematic overview of the empirical study.

In recent years, with the advent of the Internet and the explosion of web-based system development, the construct of usability has been invoked in many different ways. Research in the past decades has generated a number of procedures for assessing the usability of interactive systems. It is believed it is a multi-dimensional construct, encompassing several features. Frequently, for example, during usability inspection, there is a tendency to omit characteristics of the users, aspects of the context and peculiarities of the tasks. This tendency is reasonable and it justified by the complexity of usability as a construct and a lack of a model that unifies all of these factors. Taking into account features of users is fundamental for the User Modeling community in order to build systems that fit the specific background, knowledge and objectives of users [1–3]. Similarly, considering the context of use has a significant influence in the inference of meaningful assessments of usability [4–7]. Additionally, during the usability inspection process, accounting for the demands of the underlying task is essential for predicting user experience and informing the design of interactive systems [8, 9]. Building a cohesive model that incorporate user, context and task-specific factors is not obvious. Usability inspection should be accompanied by the assessment of one of all of these factors when possible. Beside Usability, another construct has a long research history in the field of Human Factors: the construct of human *mental workload* (MWL) [10, 11]. This is often referred to as cognitive load and I believe this can significantly contribute to the goal of informing interaction and web-design. MWL, with roots in Psychology, has been mainly adopted within the fields of Ergonomics with several application in the transportation and nuclear industries [12]. Its assessment is fundamental for predicting performance, which in turn is key for describing user experience and engagement. The link usability and mental workload is nowadays under explored. A few studies have attempted to apply the construct of MWL to explain usability [13–18]. Despite this weak interest, not much has yet been done to explore their relationship empirically. The aim of this research is to empirically investigate the relationship between subjective perception of usability and mental workload with a particular focus on their impact on objective user performance, this being assessed through observation of tangible facts. Fig 1 depicts the main constructs employed in this research study and their relationship.

The remainder of this paper is divided into the following sections. Firstly, noteworthy definitions of usability and mental workload are provided, followed by an overview of the assessment techniques employed in Human-Computer Interaction (HCI). Related work at the intersection of usability and mental workload is presented, describing how the two constructs have been employed individually and conjointly. An empirical experiment is subsequently defined in the context of human-web interaction. This is aimed at exploring the relationship between the perception of usability of three popular web-sites (youtube, wikipedia and google) and the mental workload experienced by human participants after engaging with them. Results are presented and critically examined, demonstrating how these constructs are related and how they influence objective user performance. Findings are strengthen by measures of the reliability of the underlying instruments adopted. A summary concludes this paper suggesting future research, highlighting the contribution to knowledge and implications to the field of Human-Computer Interaction.

## 1 Materials and methods

Widely employed in the broader field of HCI, usability and mental workload are two constructs from the discipline of Human Factor, with no limpid and broad definitions. Since their inception, there has been an intense debate about their assessment and measurement [19–21]. Although multidimensional and complex, their usefulness for describing the user experience and informing interaction, interface and system design is beyond doubt.

### 1.1 Usability

The amount of literature covering definitions of usability, its frameworks and methodologies is significant [22–26]. An early definition by Eason [27] was ‘the degree to which users are able to use the system with the skills, knowledge, stereotypes and experience they can bring to bear’. This definition is mainly connected to the ease-of-use, however, there are more factors influencing usability. For example, a definition provided by the ISO (International Organisation for Standardisation), is ‘The extent to which a product can be used by specified users to achieve specified goals with effectiveness, efficiency, and satisfaction in a specified context of use [28]’ (ISO 9241 series, Section 8.1). It is not a single, one-dimensional property of a user interface, rather it is often associated with the functionalities of a product rather than being merely a feature of the user interface [29]. Usability, according to Nielsen [30], is a method for improving ease-of-use in the design of interactive systems and technologies. It embraces other concepts such as efficiency, learnability and satisfaction. Nielsen’s principles are frequently employed to evaluate the usability of interfaces [30]. The evaluation is an iterative process in which usability issues are systematically discovered through the application of his principles [29]. The main limitation associated to these principles is that they are focused on the user interface under examination, overlooking contextual factors, the cognitive and emotional state of the users at the time of usability assessment as well as the complexity or time-pressure of the underlying tasks.

Often when selecting an appropriate usability assessment instrument, it is desirable to consider the effort and expense that will be incurred in collecting and analysing data, as in the context of interaction and web-design. Designers are inclined to adopt subjective usability assessment techniques for rapidly collecting feedback from users [25]. On one hand, these self-reporting techniques can only be administered post-task, thus their reliability is under discussion when done on long tasks. Meta-cognitive limitations can also mitigate the accuracy of self-reporting and thus it is challenging to perform comparisons of different raters adopting an absolute scale. On the other hand, these techniques have demonstrated their appeals because of their sensitivity and their diagnostical capacity [25]. One of this technique is the System Usability Scale [31], a questionnaire that consists of ten questions (Table A1 in S1 Appendix). It is a highly cited usability assessment technique [32]. It is a very easy scale to administer, massively applied in different domaind, showing reliability to distinguishing usable and unusable systems and interfaces both with small and large sample sizes [33–35]. Other self-reporting usability assessment techniques include the Questionnaire for User Interface Satisfaction (QUIS) [36], developed at the HCI lab at the University of Maryland, the Computer System Usability Questionnaire (CSUQ) and the Perceived Usefulness and Ease of Use scale (PUEU), both developed at IBM [37, 38]. The former was developed to assess the satisfaction of users with aspects of a computer interface [36]. It includes a demographic questionnaire, a measure of system satisfaction along six scales, as well as a hierarchy of measures of nine specific interface factors. Each of these factors relates to a user’s satisfaction with that particular aspect of an interface and to the factors that make up that facet, on a 9-point scale. The latters are a survey that consists respectively of of 19 questions on a seven-point Likert scale of ‘strongly disagree’ to ‘strongly agree’ [37] and 12 questions, from ‘extremely likely’ to ‘extremely unlikely’ [38]. Although it is more complex than other instruments, QUIS has shown high reliability across several interfaces [39]. Additional usability scales include the Purdue Usability Testing Questionnaire [40] containing 100 questions, the USE questionnaire [41], formed upon 30 questions [41]. Many other usability measures have been proposed and the reader is referred to [25, 35]. Eventually, a recent study suggests that despite the intensive use of the construct of usability in Human-Computer Interaction research, its usefulness to HCI theories as well as our understanding has been meager [42].

### 1.2 Mental workload

Human Mental Workload (MWL) is a design concept fundamental for exploring the interaction of people with technological devices, interfaces and systems [10, 43, 44]. This construct has a long history in Psychology with several applications in Human Factors, in domains such as transportation [9, 45, 46], safety-critical environments [47, 48], automation and manufacturing [49, 50], medicine and health-care [51, 52]. The principal reason for assessing mental workload is to quantify the cost associated to performing a cognitive task for predicting operator and/or system performance [53, 54]. It has been extensively documented that mental underload and overload can negatively influence performance [55]. On one hand, during information processing, when MWL is at a low level, humans may feel frustrated or often annoyed. On the other hand, when MWL is at a high level, high level of confusions can be reached by individuals with a consequent decrement in their performance while processing information and thus higher chances of making mistakes. Hence, designers who are involved in assessing human or system performance require clues about operator workload at all stages of system design and operation. These clues allow them to explore and evaluate additional design options [9]. On one hand, the difficulty of typical tasks executed on early-stage interactive systems might be initially high, due, for instance, to interface complexity. This is likely to impose high level of mental workload upon operators and thus making them experience low levels of performance [54]. This is translated in higher operator’s response time, more errors and fewer tasks are completed per unit of time [56]. On the other hand, early-stage interactive systems might be designed with simplicity in mind, initially shaping typical tasks that are likely to impose low levels of mental workload upon humans. This situation should be avoided too as it leads to difficulties in maintaining attention and promote increment in reaction time [54] with consequences on user engagement and experience. In summary, at an early design phase, a system/interface can be optimised taking mental workload into consideration, guiding designers in making appropriate structural changes [55].

MWL is not a simple and linear concept. Intuitively, it can be described as as the total cognitive work necessary for a human to accomplish a task over time [57]. It is believed that is is not ‘an elementary property, rather it emerges from the interaction between the requirements of a task, the circumstances under which it is performed and the skills, behaviours and perceptions of the operator’ [9]. This definition is merely practical, and many other factors play a role in mental workload variation. Formalising mental workload as a clear, linear construct is far from being trivial [11, 58–62] The area of MWL measurement is as extensive as its several definitions and formalisations. Several assessment techniques have been proposed in the last fifty years. Researchers in applied domains have shaped a tendency towards the use of ad hoc, domain-dependent measure or pool of measures. This trend is justified by the multi-dimensional nature of mental workload. Several reviews attempted to organise the significant amount of knowledge behind measurement procedures [10, 55, 63]. However, three main clusters are believed, by the community of MWL, to represent the main measures [54, 64–67]:

- *self-assessment or self-reporting measures*;
- *task measures or objective performance measures*;
- *physiological measures*.

The class of *self-assessment measures* is often referred to as self-report measures. This category relies upon the subject perceived experience of the interaction with an underlying interactive system through a direct estimation of individual differences such as attitudes, emotional state and level of stress of the operator, the effort devoted to the task and its demands [9, 46, 68–70]. It is strongly believed that only the human involved with a task can provide accurate and precise judgements about the MWL experienced. For this reason, self-assessment measures have been always appealing to many practitioners. The class of task *performance measures* is based on the assumption that the mental workload of an individual, interacting with an underlying system or interface, becomes relevant only if it impacts system performance. Example of measures include reaction time to a secondary task, task completion time, error rate, tapping regularity. In turn, this category appears as the most valuable options for designers [71–73]. The category of *physiological measures* considers responses of the body gathered from the individual interacting with an underlying task and system. These responses are thought to be highly correlated to MWL. Their utility lies in the interpretation and analysis of psychological processes and their effect on the state of the body. Example of these measures include brain function measures, cardiac measures such as hear-rate, eye measures, such as pupil dilation/movement and muscle measures. The advantage behind measures belonging to this category is that they can be collected continuously over time, without demanding an explicit response by the operator [74, 75]. So far they have required specific equipment and trained operators to employ this equipment minimising their employability in real-world tasks [76]. However, this tendency is assisting to a shift thanks to the advances in sensor-based technologies to monitor physiological signals.

#### 1.2.1 A focus on self-assessment measures

Self-assessment measures of mental workload have in general low implementation requirements, they are often not intrusive and possess high degree of acceptability by end-users [69, 77]. These measures are usually multi-dimensional. Examples include the NASA’s Task Load Index (*NASATLX*) [9], the Subjective Workload Assessment Technique [70] and the Workload Profile [77]. Uni-dimensional measures also exist such as the the Rating Scale Mental Effort [78], the Copper-Harper scale [79], the Bedford scale [80] and the Subjective Workload Dominance Technique [81]. Among this, the *NASATLX* is probably the most popular self-reporting MWL technique [9]. This has been used in many empirical studies as for instance, to evaluate user interfaces in health-care or in e-commerce application and for the improvement of user satisfaction [82]. [83] investigated how the design of query interfaces is related to performance and stress during information-seeking tasks. Mental workload was assessed using the *NASATLX* and log data was used as objective indicator of performance to characterise searching behaviour. The Workload Profile [77], the *NASATLX* and the Subjective Workload Assessment Technique [70] have been compared in a user study to evaluate different web-based interfaces [84]. In general, these techniques have demonstrated a good internal reliability (Cronbach’s Alpha varying around.80) and external validity [85]. In this research study, the Nasa Task Load Index and the Workload profile techniques have been adopted. These self-reporting techniques are described in details respectively in section 1.4.2 and section 1.4.3.

### 1.3 Research at the intersection of usability and mental workload

Not a lot of research exist at the intersection of mental workload and usability. O’Brien and collaborators identified mental workload and usability as dimensions of the construct of user engagement, showing these are weakly correlated to each other [15]. A similar view can be found in the proposal presented in [86]. Lehmann et al. highlighted the usefulness of adopting different metrics for assessing user engagement, such as usability and cognitive engagement [14]. In a recent review, it was acknowledge that usability and performance are two key elements for describing user experience [16]. The above work clearly emphasises the usefulness of adopting mental workload with traditional usability assessment methods for explaining user experience and user engagement. The constructs of mental workload and usability have been jointly mentioned in an article to better design e-learning artefacts in medical education [87]. Nonetheless, the above contributions are mainly theoretical with little empirical value. Tracy and Albers attempted to use the construct of mental workload to test the usability of web-sites [17] and allowing the identification of those high-workload sub-areas of the interface that required attention. A similar study was aimed at investigating tapping as a measure of mental workload and website usability [13]. Gahangir et al. attempted to understand the convergence of usability and cognitive load in evaluating the performance of fully integrated assistive technology solutions [88] when adopted by blind people. Their study showed a high correlation of a secondary task performance measure, and three types of load (intrinsic, germane and extraneous) treated as cognitive load indexed, to usability, measured with a modification of some of the dimensions proposed in [89]. Similarly, another study employed an index of mental workload, namely the NASA-TLX, in conjunction with a measure of usability, namely the USE questionnaire for evaluating an interface for social robotic telepresence [90]. Unfortunately, the sample size of the data employed in the above empirical studies is not enough to draw any credible conclusion about the interaction mental workload-usability.

Despite research at the intersection of mental workload and usability is sparse and limited, a number of papers have been published discussing the relationship between usability and task performance. In [91], preference, a parameter of usability, and human performance were positively associated. However, it pointed out the existence of cases where users would not prefer an underlying interactive system whose design would seem more aligned to objective performance measures. This line of thought also appeared in the meta-analysis conducted in [92] over 73 usability studies. Here authors suggest that measures of users? perceptions of phenomena, in general, are not correlated with objective measures of the phenomena.

### 1.4 Design of experiments

An empirical study with human participants has been designed and executed. Users had to interact with 3 popular web-sites (youtube, google, wikipedia) and execute typical tasks over them (Table A5 in S1 Appendix). The aim was to explore the relationship between the perception of usability, the subjective mental workload experience by users and the achieved objective performance. One self-reporting procedure for measuring usability and two self-reporting methods for mental workload assessment have been selected:

- the System Usability Scale (*SUS*) [31]
- the Nasa Task Load Index (*NASATLX*), developed at NASA [9]
- the Workload Profile (*WP*) [77], based on Multiple Resource Theory [93, 94].

**Fig 2:**
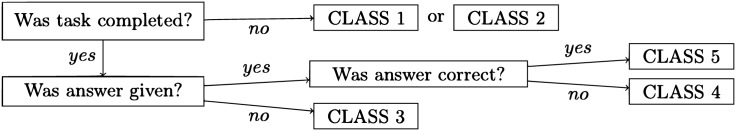
Partial dependencies of objective performance classes.

**Fig 3:**
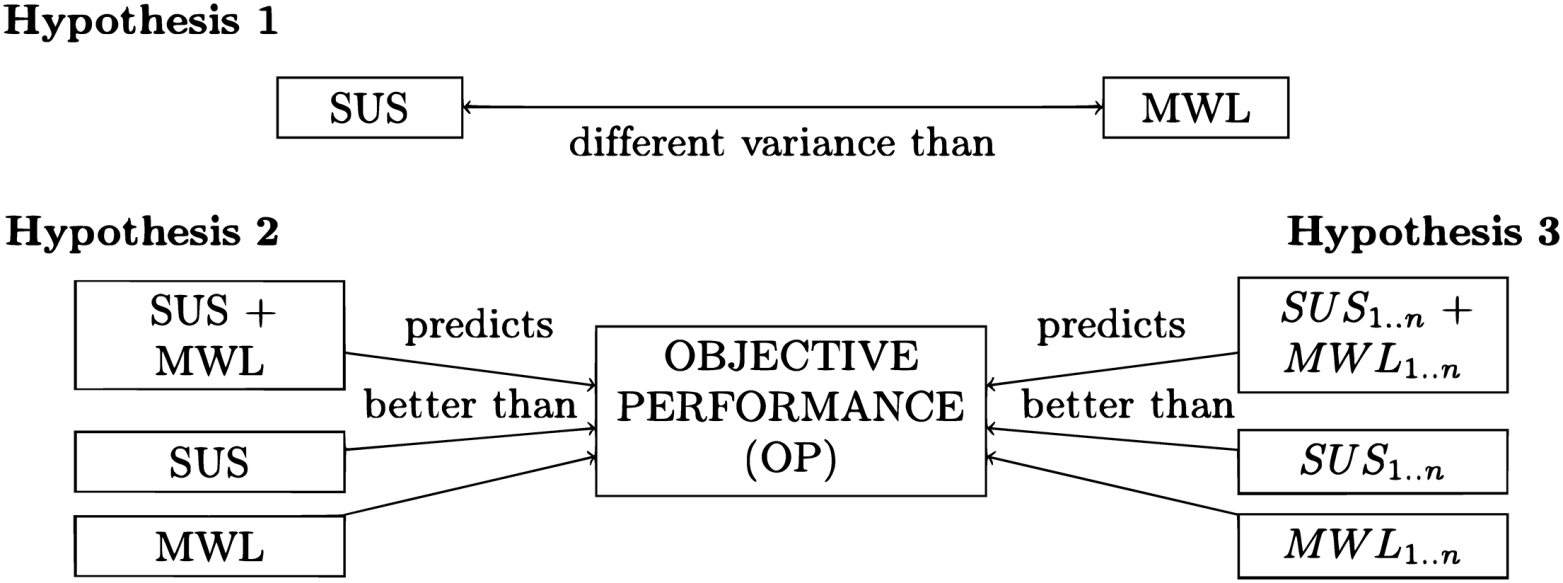
Illustration of research hypotheses.

No physiological procedures for mental workload measurement were included in the study. This was not considered because the goal of this research is to investigate the relationship between perception of usability, through a questionnaire, and assessment of mental workload with self-reporting measures. Five classes of user objective performance on tasks have been set (Table 1). These classes of objective performance are sometimes conditionally dependent (Fig 2) and the associated number (1–5) is not an indication of their strength or rank but merely a label. The detailed research hypotheses are defined in Table 2 and illustrated in Fig 3. The three measurement techniques are detailed below, followed by the definition of the research hypotheses.

**Table 1:** Description of objective performance classes.

Class	Description
1	the task was not completed as the user gave up
2	the execution of the task was terminated because available time elapsed
3	the task was completed and no answer was required by the user
4	the task was completed, the user provided an answer, but it was wrong
5	the task was completed and the user provided the correct answer

**Table 2:** Description of research hypotheses.

label	description
*H*_1_	Usability and Mental workload are two uncorrelated constructs capturing difference variance (as measured with self-reporting techniques—SUS, NASATLX, WP).
*H*_2_	A unified model incorporating a usability and a mental workload measure can significantly enhance the accuracy of the prediction of objective performance than the individual usability and MWL models.
*H*_3_	A hybrid model incorporating features of a measure of usability and features of a measure of mental workload can significantly enhance the prediction of objective performance than models incorporating only usability or MWL features.

#### 1.4.1 The system usability scale

The System Usability Scale is a subjective usability assessment instrument that include a Likert scale, bounded in the range 1 to 5 [31]. Questions can be found in Table A1 in S1 Appendix. Individual scores are not meaningful on their own. For odd questions (*SUS*_*i*_ with *i* = {1|3|5|7|9}), the score contribution is the scale position (*SUS*_*i*_) minus 1. For even questions (*SUS*_*i*_ with *i* = {2|4|6|8|10}), the contribution is 5 minus the scale position. For comparison purposes, the *SUS* value is converted in the range [1..100] ∈ ℜ with *i*_1_ = {1, 3, 5, 7, 9}, *i*_2_ = {2, 4, 6, 8, 10}:

$$\begin{array}{c} SUS:\left[0..100\right]\in ℜ\;\;\;\;\;SUS=\frac{1}{10}\cdot \left[\sum_{i_{1}}\left(SUS_{i}\right)+\sum_{i_{2}}\left(100-SUS_{i}\right)\right] \end{array}$$

#### 1.4.2 The NASA task load index

The Nasa Task Load Index [9] belongs to the category of self-assessment measures. It has been validated in the aviation industry and other contexts in Ergonomics [9, 71] with several applications in many socio-technical domains. It is a combination of six factors believed to influence MWL (questions of Table A2 in S1 Appendix). Each factors is quantified with a subjective judgement coupled with a weight computed via a paired comparison procedure. Subjects are required to decide, for each possible pair (binomial coefficient, (62)=15) of the 6 factors, *‘which of the two contributed the most to mental workload during the task’*, such as ‘Mental or Temporal Demand?’, and so forth. The weights *w* are the number of times each dimension was selected. In this case, the range is from 0 (not relevant) to 5 (more important than any other attribute). The final MWL score is computed as a weighed average, considering the subjective rating of each attribute *d*_*i*_ and the correspondent weights *w*_*i*_:

$$\begin{array}{c} NASATLX:\left[0..100\right]\in ℜ\;\;\;\;\;NASATLX=\left(\sum_{i=1}^{6}d_{i}\times w_{i}\right)\frac{1}{15} \end{array}$$

#### 1.4.3 The workload profile

The Workload Profile assessment procedure [77] is built upon the Multiple Resource Theory proposed in [93, 94]. In this theory, individuals are seen as having different capacities or ‘resources’ related to:

- *stage of information processing*—perceptual/central processing and response selection/execution;
- *code of information processing*—spatial/verbal;
- *input*—visual and auditory processing;
- *output*—manual and speech output.

Each dimension is quantified through subjective rates (questions of Table A3 in S1 Appendix) and subjects, after task completion, are required to rate the proportion of attentional resources used for performing a given task with a value in the range 0..1 ∈ ℜ. A rating of 0 means that the task placed no demand while 1 indicates that it required maximum attention. The aggregation strategy is a simple sum of the 8 rates *d* (averaged here, and scaled in [1..100] ∈ ℜ for comparison purposes):

$$\begin{array}{c} WP:\left[0..100\right]\in ℜ\;WP=\frac{1}{8}\left(\sum_{i=1}^{8}d_{i}\times 100\right) \end{array}$$

#### 1.4.4 Research hypotheses

Three research hypotheses have been defined in this empirical research (Table 2). The first hypothesis is a non-directional statement, anticipating that usability, as measured by SUS, captures a different variance than MWL, as measured by NASATLX or WP. It is anticipated that there is a relatively random relationship between usability and mental workload, indicating two uncorrelated constructs.

The second and third hypotheses are directional predictive statements. In detail, the second hypothesis anticipates that the usability and mental workload measures can be successfully combined together to predict objective performance better than the individual measures.

The third hypothesis assumes that the dimensions used to form a usability or a mental workload index cannot be combined together to predict the objective performance better than the individual model-specific dimensions. In other words, a hybrid model incorporating both attributes of usability and mental workload will form a new, unknown construct that does not contribute to enhance the prediction of objective performance when predicted with the attributed of the individual models. For clarification purposes, the above hypotheses are stated in formal terms in Table 3.

**Table 3:** Formal description of research hypotheses (*corr* a correlation coefficient and *acc* the accuracy of a prediction).

label	formal description
*H*_1_	a)*corr*(*SUS*, *NASATLX*) = 0 b)*corr*(*SUS*, *WP*) = 0
*H*_2_:	a) *acc*(*SUS* → *OP*) < *acc*(*SUS*, *NASATLX* → *OP*) b) *acc*(*SUS* → *OP*) < *acc*(*SUS*, *WP* → *OP*) c) *acc*(*NASATLX* → *OP*) < *acc*(*SUS*, *NASATLX* → *OP*) d) *acc*(*WP* → *OP*) < *acc*(*SUS*, *WP* → *OP*)
*H*_3_:	a) *acc*(*SUS*_1,..,10_ → *OP*) < *acc*(*SUS*_1,..,10_, *NASA*_1,..,6_ → *OP*) b) *acc*(*SUS*_1,..,10_ → *OP*) < *acc*(*SUS*_1,..,10_, *WP*_1,..,8_ → *OP*) c) *acc*(*NASA*_1,..,6_ → *OP*) < *acc*(*SUS*_1,..,10_, *NASA*_1,..,6_ → *OP*) d) *acc*(*WP*_1,..,8_ → *OP*) < *acc*(*SUS*_1,..,10_, *WP*_1,..,8_ → *OP*)

#### 1.4.5 Participants and procedure

Due to the fact that this research involved human participants, the study has been approved by the ethics committee of the University of Dublin, Trinity College where the experiment has been carried out. The study has been conducted according to the principles expressed in the Declaration of Helsinki. Participants have been properly instructed and have indicated that they consent to participate by signing the appropriate informed consent paperwork. All efforts have been made by the author to protect the privacy and anonymity of participants. Participants were recruited initially through the use of internal mailing list in the School of Computer Science and Statistics at Trinity College Dublin. However, since the response was not as expected, with a drop out rate of 80%, probably due to the length of the experiment itself, the recruitment process was extended to personal contacts of the first participants through word-of-mouth.

A sample of 46 human volunteers, fluent in English, eventually participated in the research after signing the consent form. Participants were divided into 2 groups of 23 each: those in group A were different to those in group B. Volunteers could not engage with instructors during the execution of the tasks and their trained was not required. Ages ranges from 20 to 35 years; 24 females and 22 males evenly distributed across the 2 groups (Total—Avg.: 28.6, Std. 3.98; g.A—Avg. 28.35, Std.: 4.22; g.B—Avg: 28.85, Std.: 3.70) all with a daily Internet usage of at least 2 hours. Volunteers were asked to execute a set of 9 information-seeking web-based tasks (Table A5 in S1 Appendix) in the most natural way, over 2 or 3 sessions of approximately 45/70 minutes each, on different non-consecutive days on three popular web-sites. The number of sessions were established according to their availability to participate in the research. Designed information-seeking tasks differed in terms of intrinsic complexity, time-pressure, time-limits, human interference and interruptions as well as demands on different modalities (visual, auditory, information-processing). Also, three popular web-sites were selected with the assumption that participants had previously interacted with them at least once. The rationale behind this is the expectation to observe situations of underload for more assiduous Internet users, given their familiarity with the underlying interface. Two groups were formed because designed tasks were going to be executed on original and run-time altered web-interfaces (through a CSS/HTML manipulation, as in Table A4 in S1 Appendix). The rationale behind this manipulation was to allow the formation of scenarios in which even assiduous Internet users were expected to perceive an higher mental workload. If non-popular web-sites would have been selected, the chances to spot scenarios of underload were minimal. This is because of the unfamiliarity of the users with the new web-sites and the higher effort that would have been required for the execution of the experimental tasks. Additionally, participants had to interact with those web-sites multiple times, executing different tasks one after each other. This means that, even with the same interface, complexity of tasks could have been perceived differently, given the increasing level of fatigue of participants, boredom or annoyance task after task. The run-time manipulation of web-sites was also planned as part of a larger research study [95–97], to enable A/B testing of web-interfaces (not included here). Interface manipulation was not extreme, like making things very hard to read. Rather the goal was to manipulate the original interface to alter usability and task difficulty independently. The order of the tasks administered was the same for all the volunteers. Computerised versions of the *SUS* (Table A1 in S1 Appendix), the *NASATLX* (Table A2 in S1 Appendix) and the *WP* (Table A3 in S1 Appendix) instruments were administered shortly after task completion. Note that the question of the *NASA* − *TLX* related to the ‘physical load’ dimension was set to 0 as well as its weight as no physical effort was required. As a consequence, the resulting pairwise comparison procedure became shorter. Some participant did not execute all the tasks and the final dataset contains 390 cases.

## 2 Results

**Fig 4:**
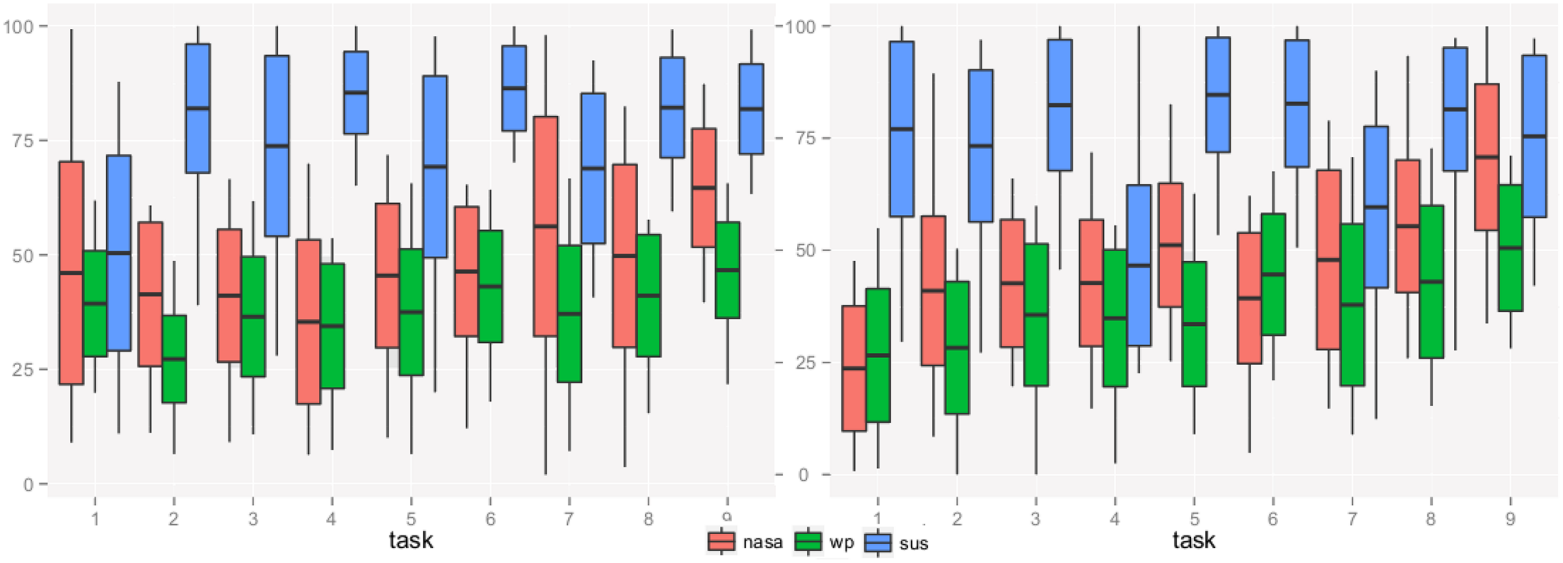
Summary statistics by task.

Table 4 and Fig 4 show the means and the standard deviations of the usability and the mental workload scores for each information-seeking task (Table A5 in S1 Appendix).

**Table 4:** Mental workload & usability—Groups A, B (G.A/G.B).

G. A	*NASATLX*		*WP*		*SUS*	
Task	avg	std	avg	std	avg	std
1	46.03	24.30	39.34	11.54	50.38	21.31
2	41.38	15.71	27.23	9.51	81.98	14.06
3	41.08	14.47	36.50	13.10	73.77	19.71
4	35.36	17.92	34.43	13.61	85.41	8.96
5	45.47	15.74	37.49	13.78	69.22	19.84
6	46.35	14.13	43.09	12.20	86.36	09.26
7	56.20	23.97	37.11	14.92	68.87	16.38
8	49.76	19.96	41.09	13.31	82.16	10.93
9	64.61	12.92	46.65	10.46	81.85	09.81
G. B	*NASATLX*		*WP*		*SUS*	
Task	avg	std	avg	std	avg	std
1	23.66	13.93	26.57	14.85	77.00	19.49
2	40.97	16.62	28.27	14.73	73.24	16.92
3	42.63	14.21	35.60	15.81	82.33	14.58
4	42.70	14.09	34.87	15.25	46.61	17.90
5	51.15	13.78	33.54	13.88	84.64	12.77
6	39.31	14.57	44.61	13.50	82.68	14.12
7	47.86	19.97	37.84	18.02	59.62	17.97
8	55.34	14.75	42.97	16.98	81.41	13.73
9	70.75	16.29	50.51	14.06	75.39	18.02

### 2.1 Testing hypothesis 1

**Fig 5:**
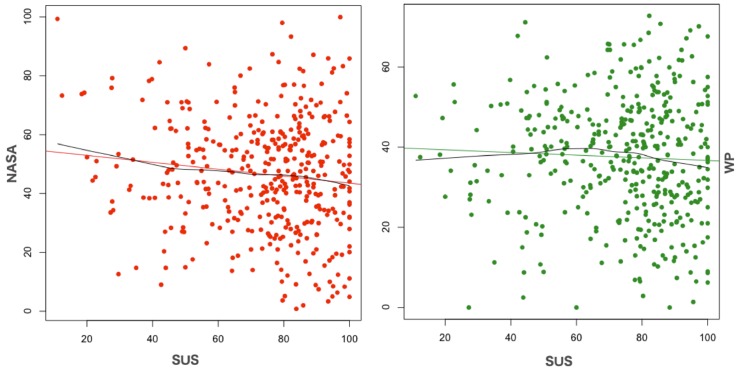
Scatterplots of *NASATLX*, *WP* vs *SUS*.

To test hypothesis 1, a correlation analysis of the usability versus the mental workload scores has been performed. From an initial analysis of the data depicted in Fig 5, it seems intuitive to assess a random relationship between the usability scores (*SUS*) and the mental workload scores (*NASATLX*, *WP*). This is statistically confirmed in Table 5 by the Pearson and Spearman correlation coefficients computed over the full dataset (Groups A, B). The Pearson coefficient was chosen for exploring a linear correlation between the two constructs while the Spearman correlation for investigating the existence of a monotonic relationship, not necessarily linear.

**Table 5:** Pearson and Spearman correlation coefficients of the usability and the mental workload scores.

	Pearson		Spearman	
	*WP*(p-val)	*SUS*(p-val)	*WP*(p-val)	*SUS*(p-val)
*NASA*	0.55(<.001)	-0.13(.007)	0.53(<.001)	-0.1(.03)
*WP*		-0.05(.35)		-0.08(.11)

**Fig 6:**
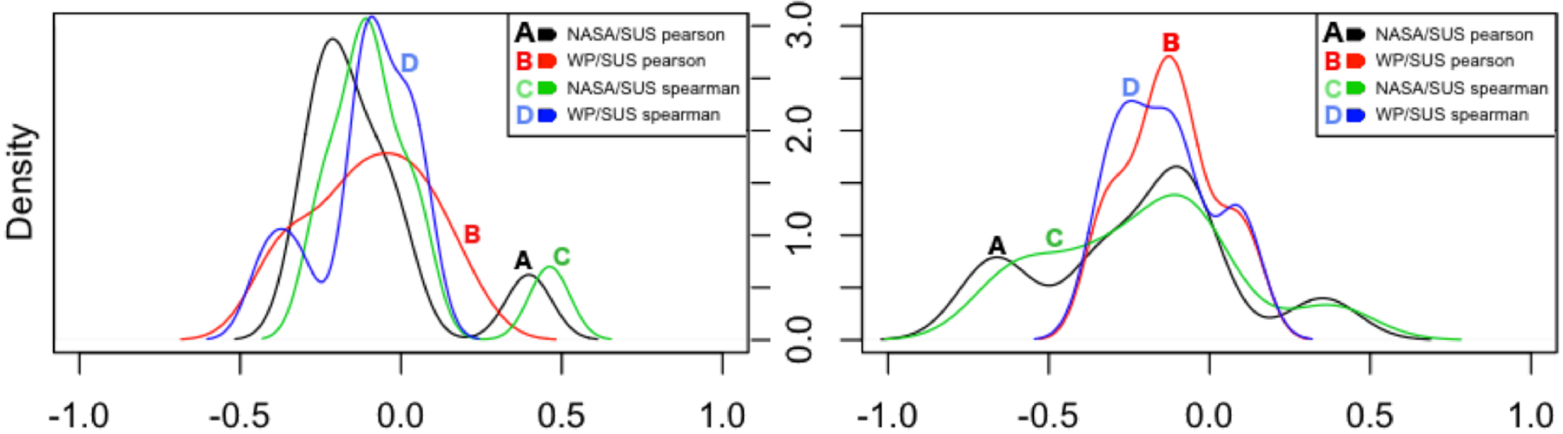
Density plots of the correlations by task—Group A, B.

Although the perception of usability does not seem to correlate at all with the subjective mental workload experienced by participants, a further investigation of their relationship was performed on a task-basis. Fig 6 depicts the density plots of the correlations achieved between the usability and mental workload scores, while Table 6 formally list their magnitude. Note that each density function contains 9 values, one for each task. Additionally, although the description of the tasks was identical across groups, participants executed them over two difference interfaces, therefore tasks in group A where considered different than tasks in group B. Commonly, in behavioural and social sciences, there may be a greater contribution from complicating factors, as in the case of subjective, self-reported ratings. Therefore, correlations above 0.5 are regarded as very high, within [0.3 − 0.5] as medium/moderate and within [0.1 − 0.3] small (symmetrically to negative values) [98](page 82). In this analysis, only medium and high correlation coefficients are taken into account (highlighted in Table 6), discarding those demonstrating random relationship.

**Table 6:** Correlations MWL vs usability. Groups A and B.

G. A	Pearson		Spearman	
Task	Nasa/SUS	WP/SUS	Nasa/SUS	WP/SUS
1	-0.21	-0.39	-0.24	-0.42
2	-0.22	0.18	-0.1	0.01
3	-0.25	-0.13	-0.23	-0.08
4	-0.05	-0.11	-0.10	-0.09
5	0.14	-0.26	0.10	-0.27
6	-0.17	-0.01	0.04	0.06
7	-0.11	0.03	-0.10	0.03
8	-0.28	0.02	-0.13	-0.13
9	0.48	-0.15	0.57	-0.15
G. B	Pearson		Spearman	
Task	Nasa/SUS	WP/SUS	Nasa/SUS	WP/SUS
1	-0.69	-0.06	-0.6	-0.11
2	-0.12	-0.15	-0.15	-0.23
3	-0.07	0.13	-0.05	0.11
4	-0.64	-0.34	-0.60	-0.34
5	-0.34	-0.08	-0.31	-0.08
6	-0.08	-0.14	-0.07	-0.12
7	-0.32	-0.2	-0.37	-0.30
8	-0.08	-0.29	-0.04	-0.24
9	0.36	0.14	0.44	0.14

**Fig 7:**
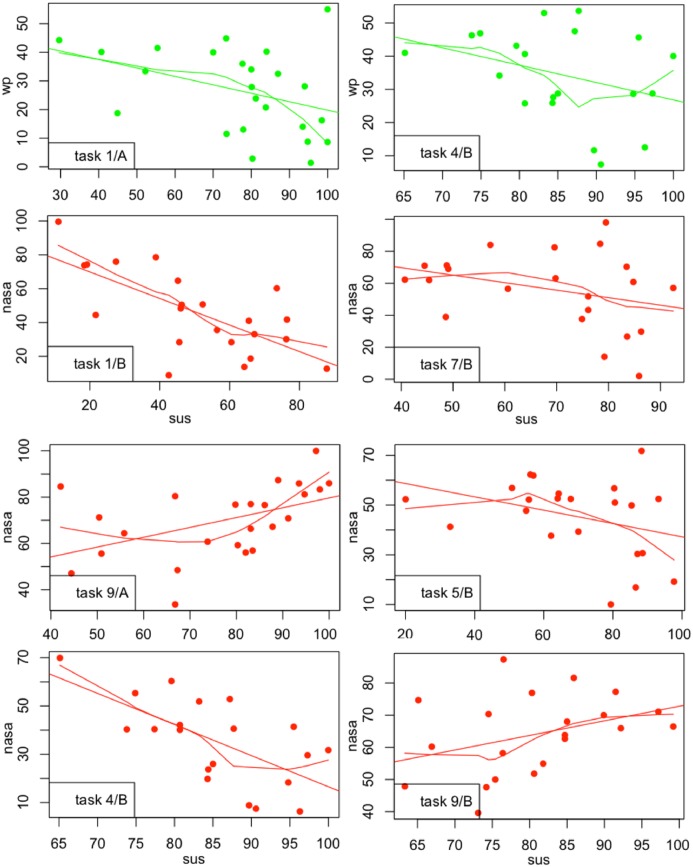
Details of tasks with moderate/high correlation.

Yet, a clearer picture does not emerge and just for a few tasks some form of relationship exists between perception of usability and the mental workload experienced by participants. Fig 7 is aimed at visually help with the explanation of these cases, extract further information and possible interpretations on why the usability scores were moderately or highly correlated with the workload scores.

- tasks 1/A and 4/B: *WP* scores seem to be moderately negatively correlated with the *SUS* scores. This might suggest that *when the proportion of attentional resources being taxed by a task is moderated and decreases, the perception of good usability increases*. In other words, when web-interfaces and the tasks executed over them require a moderate use of different stages, codes of information processing and input, output modalities (section 1.4.3), the usability of those interfaces is increasingly perceived as positive.
- tasks 9/A and 9/B: the *NASATLX* scores are highly and positively correlated with the *SUS* scores. This might suggests that, even when time pressure is imposed upon tasks (description of task 9 in Table A5 in S1 Appendix) causing an increment in the workload experienced, and the perception of performance decreases because the answer of the task is not found, than the perception of usability is not affected if the task is pleasant and amusing (like task 9). In other words, *even if the experienced mental workload increases but not excessively, and even if an interface is slightly altered (task 9 group B), the perception of positive usability is strengthened if tasks are enjoyable*.
- tasks 1/B, 4/B, 5/B, 7/B: the *NASATLX* scores are highly negatively correlated with the *SUS* scores. This might suggests that *when the mental workload experienced by users increases, perhaps because tasks are not straightforward, the perception of usability can be negatively influenced even with a slight alteration of the interface*.

**Fig 8:**
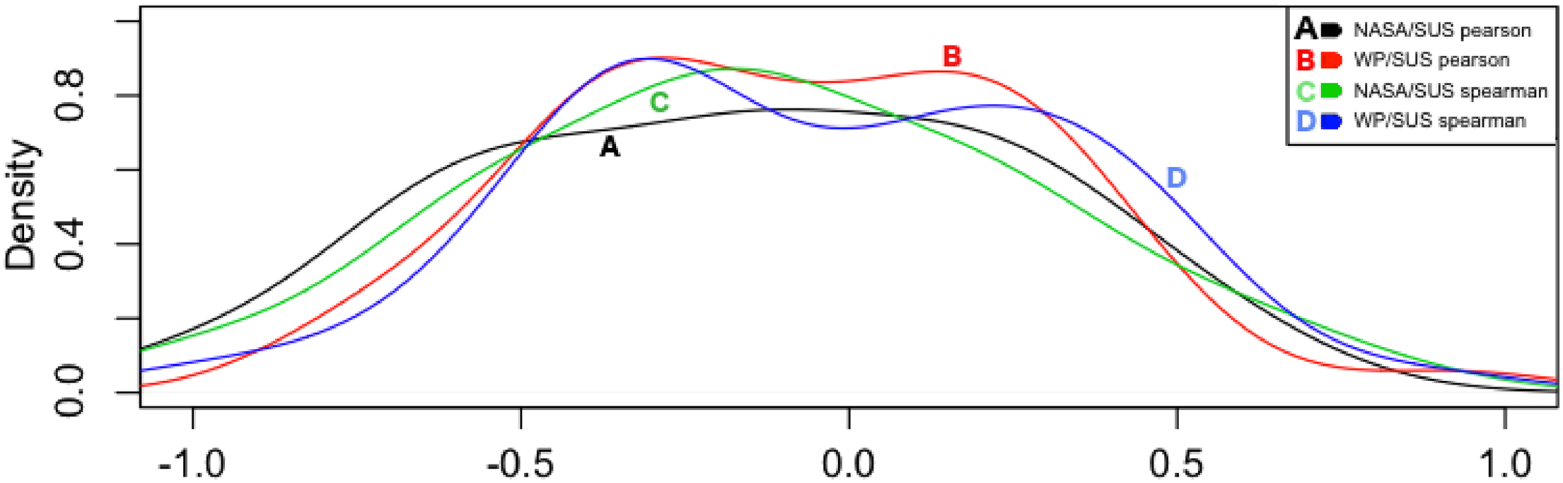
Density plots of the correlations by user.

The above interpretations do not aim to be exhaustive. Rather they are possible interpretations and are only confined to this study. Further empirical investigations are required prior to generalising these findings. To further strengthening the data analysis, an investigation of the correlation between the usability and the mental workload scores has been performed on a participant-basis (Table 7 and Fig 8).

**Table 7:** Correlation MWL-usability by user.

	Pearson		Spearman	
User	Nasa/SUS	WP/SUS	Nasa/SUS	WP/SUS
1	-0.5	-0.43	-0.45	-0.32
2	0.41	-0.11	0.57	-0.23
3	-0.4	0.18	-0.27	0.45
4	0.38	0.37	0.15	0.17
5	-0.66	-0.57	-0.7	-0.63
6	-0.15	-0.34	-0.06	-0.14
7	-0.17	-0.2	-0.17	-0.4
8	-0.23	0.13	-0.54	0.01
9	-0.16	-0.4	-0.25	-0.08
10	0	0.26	-0.05	0.33
11	-0.47	-0.74	-0.52	-0.78
12	0.64	-0.3	0.61	-0.34
13	-0.17	0.18	-0.23	0.18
14	0.24	0.39	-0.22	0.16
15	0.06	0.17	0.21	0.47
16	0.46	0.34	0.57	0.55
17	0.27	0.02	0.15	0.23
18	-0.14	0.16	-0.15	-0.2
19	-0.76	0.05	-0.55	-0.03
20	0.05	-0.21	0.27	0.18
21	0.43	-0.06	0	0.1
22	-0.99	0.05	-1	0.4
23	0.18	-0.2	0.4	-0.33
24	0.19	0.32	-0.25	0.19
25	-0.62	-0.07	-0.38	-0.4
26	-0.69	0.29	-0.62	0.38
27	-0.38	-0.36	-0.55	-0.58
28	-0.13	-0.43	-0.2	-0.48
29	-0.11	0.28	-0.03	0.15
30	0.17	-0.22	0.22	-0.38
31	-0.6	-0.42	-0.78	-0.48
32	-0.7	-0.4	-0.2	-0.22
33	0.06	-0.67	0	-0.32
34	-0.41	-0.45	-0.32	-0.27
35	0.19	-0.08	0	0.08
36	-0.34	-0.15	-0.58	-0.48
37	-0.47	-0.08	-0.17	0.38
38	0.21	0.43	0.32	0.51
39	-0.17	-0.07	0.2	0.12
40	-0.34	0.93	0.1	0.87
41	0.25	-0.23	0.37	-0.35
42	-0.67	-0.6	-0.65	-0.38
43	0.02	0.18	-0.07	-0.04
44	-1	-0.79	-1	-1
45	-0.59	-0.36	-0.4	-0.23
46	0.27	0.53	0.21	0.34

**Fig 9:**
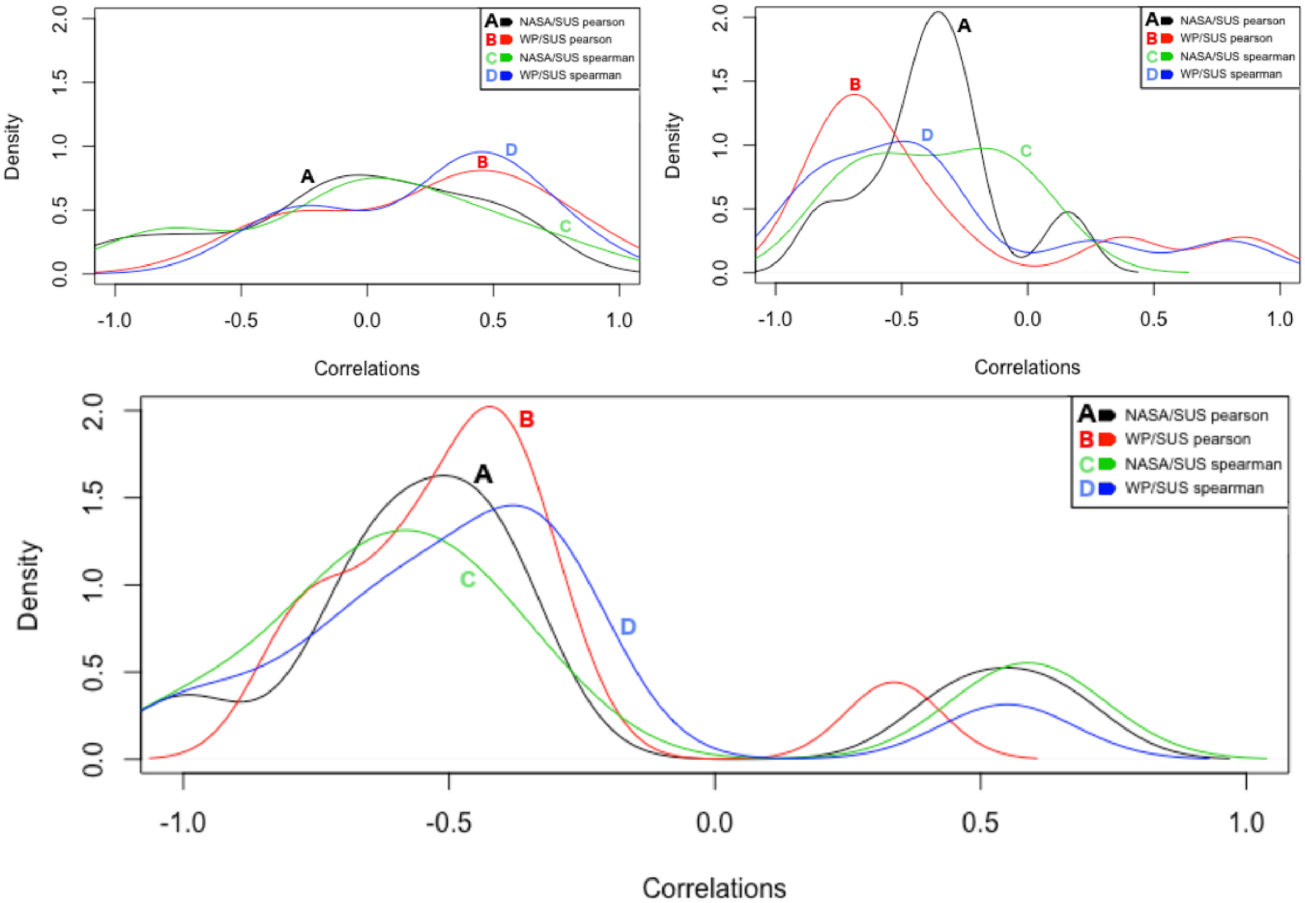
Density plots of the correlations of selected users by task (top, groups A, B) and by users (bottom).

As in the previous analysis (by task), just medium and high correlation coefficients (>0.3) are taken into account for a deeper exploration. Additionally, because the correlations listed in Table 6 were not able to systematically demonstrate common trends, the analysis on the individual-basis was more strict. In details, only those scores of participants for which a medium or high linear relationship (Pearson) and a monotonic relationship (Spearman) was found between both the two MWL indexes (*NASA*, *WP*) and the usability scores (*SUS*) was taken into consideration. The goal was to look for the presence of any peculiar pattern of user’s behaviour or a more complex deterministic structure. These participants are highlighted in Table 7 (1, 5, 11, 12, 16, 27, 31, 42, 44). The densities of their correlations are depicted in Fig 9.

**Fig 10:**
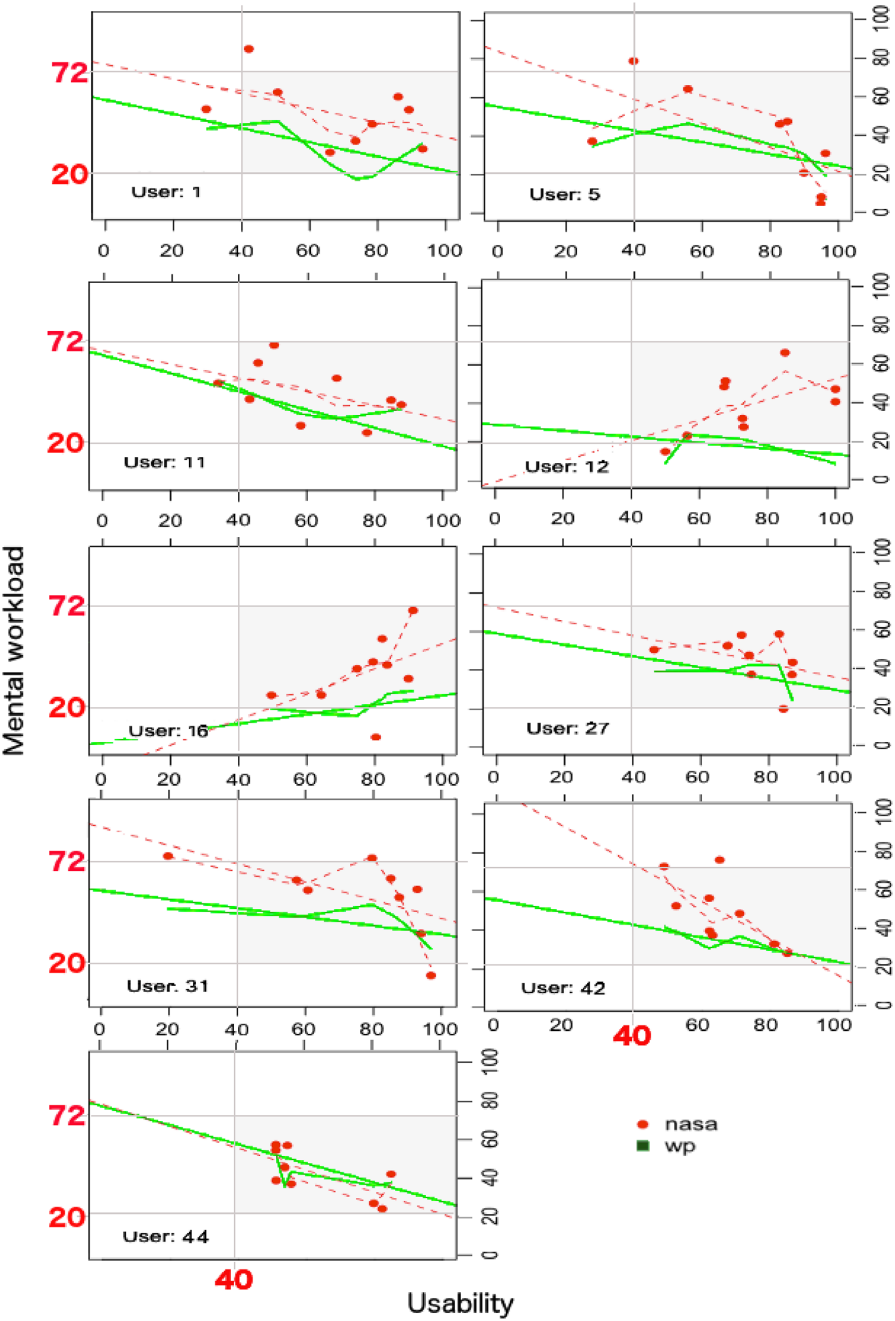
Relationship between usability and mental workload scores for participants with moderate or high Pearson and Spearman correlation coefficients.

From Fig 8 (bottom), a multimodal distribution of the correlations of the usability and mental workload scores emerges, with a big cluster of users close to −0.5 and a smaller one close to 0.5. Fig 10 show the linear scatterplots associated to these participants with a linear straight regression line and a local smoothing regression line (Lowess algorithm [99]). The former type of linear regression is parametric and assumes normal distribution of data, while the latter is non-parametric, it does not necessarily assumes normality of data and it aids the identification of patterns, increasing the ability to see a line of best fit over data. Outliers from scatterplots have not been removed due to the limited available points—9 points which are the maximum tasks executed by each participant.

Yet, no relational pattern between perception of usability and subjective mental workload clearly emerges. Nonetheless, by reviewing the mental workload scores (*NASATLX* and *WP*), it appears that the nine selected participants have all experienced, except a few outliers, optimal mental workload (on average between 20 and 72). These users did not perceive situations of underload or overload while executing the underlying tasks. Similarly, taking a closer look at the usability scores, these users did not perceive an extremely negative usability, with scores higher than 40. Except users 12 and 16, all the others seem to share a property: the lower the experienced mental workload is when between optimal ranges, the lower their perception of usability.

A final attempt to investigate the relationship between experienced workload and perceived usability has been performed by grouping scores by the objective performance class. Table 8 lists the correlations of the workload and usability scores by performance class.

**Table 8:** Correlations of the mental workload scores with the usability scores by performance class.

	Pearson		Spearman	
Class	NASA vs SUS	WP vs SUS	NASA vs SUS	WP vs SUS
1	-0.09	-0.14	-0.14	-0.26
2	0.08	-0.32	0.16	-0.24
3	-0.13	0.06	-0.04	-0.10
4	0.15	0.09	0.09	-0.02
5	-0.17	-0.02	-0.14	-0.03

**Fig 11:**
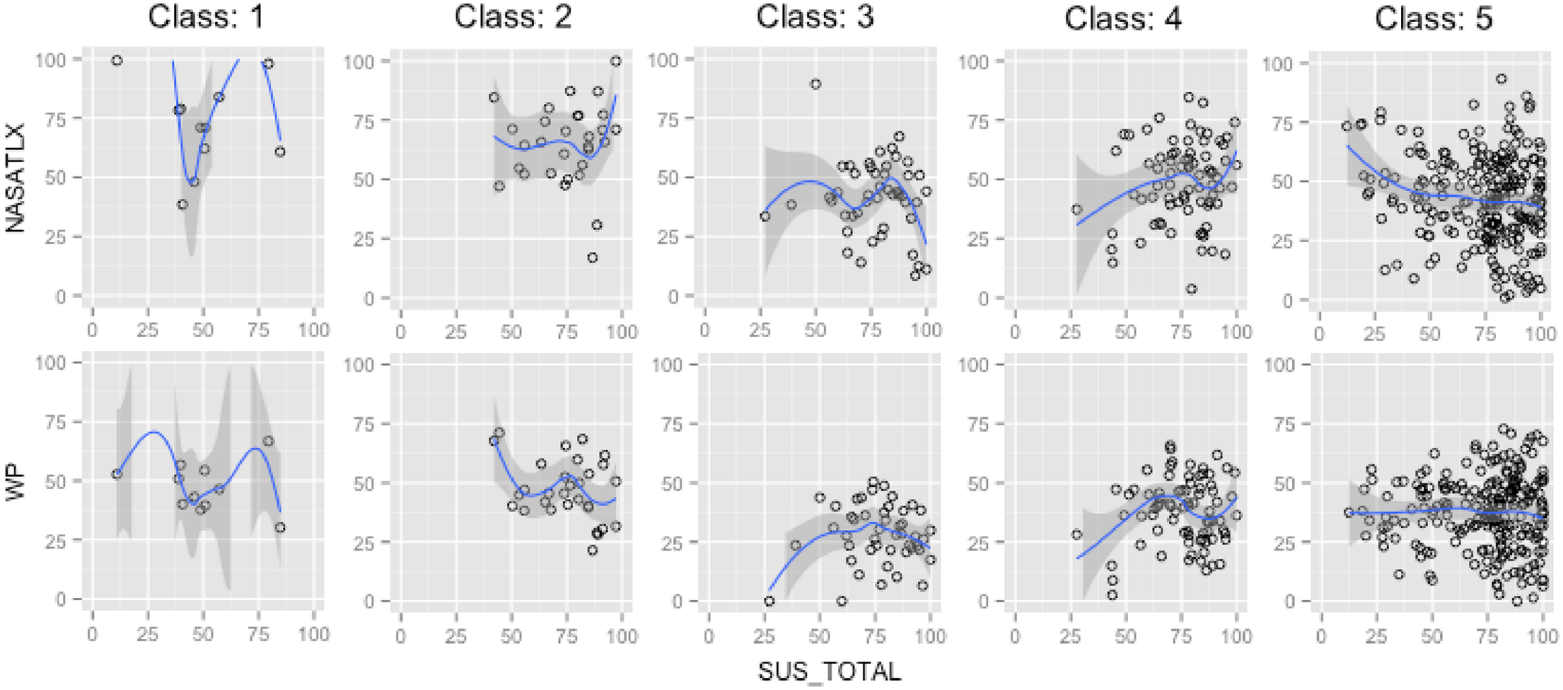
Correlations of the mental workload scores and the usability scores by performance class.

Unfortunately, no trend emerges from Table 8 and Fig 11 with the correlations between experienced mental workload and perceived usability all close to zero, suggesting a random relationship between the two constructs.

### 2.2 Testing hypothesis 2

**Fig 12:**
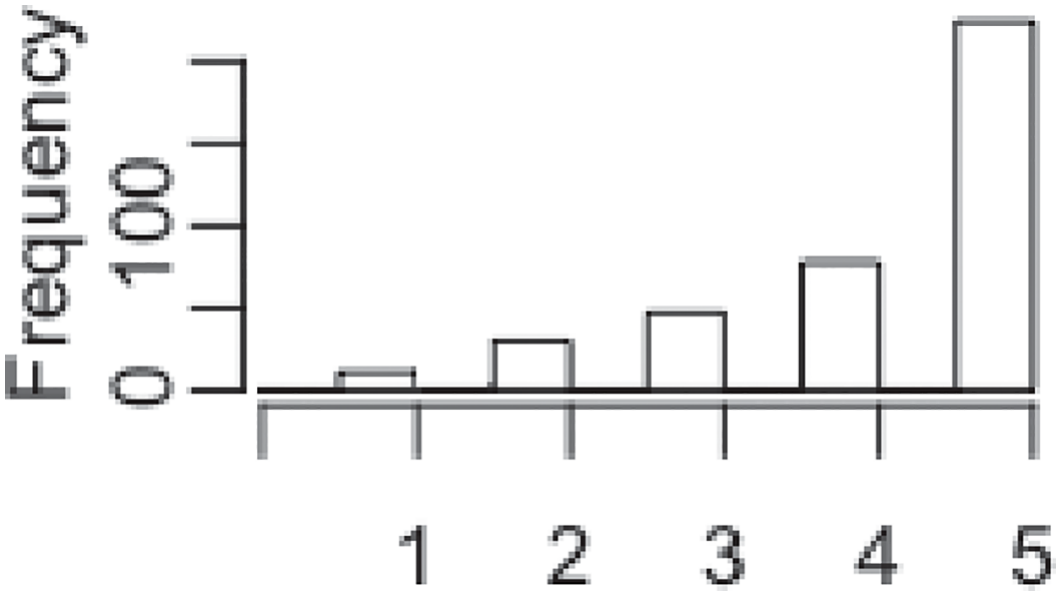
Original distribution of the objective performance classes.

In order to test hypothesis two, an investigation of the impact of the perception of usability and the experienced mental workload on the objective performance achieved by user has been conducted. In this context, objective performance refers to objective indicators of the performance reached by each volunteers who participated in the user study, categorised in 5 classes (section 1.4). During the experimental study, the measurement of the objective performance of some user was faulty. These cases were discarded and a new dataset with 390 valid cases was formed. The investigation of the impact of the perception of usability and the mental workload experienced by users on the 5 classes of objective performance was treated as a classification problem, employing supervised machine learning. The distribution of these five classes are depicted in Fig 12 and Table 9.

**Table 9:** Frequencies of classes.

Class	Original	Oversampled
1	11	224
2	30	224
3	47	224
4	78	224
5	224	224
total	390	1120

**Fig 13:**
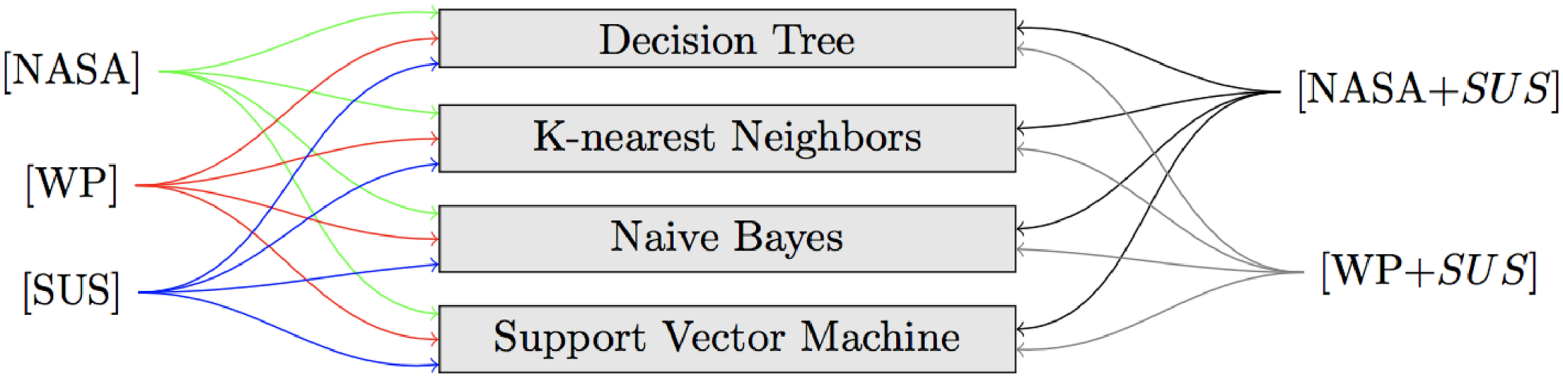
Independent features and classification techniques.

Clearly, the original class frequencies are unbalanced and this is likely to have a negative influence on the classification of the performance classes. To mitigate this risk, a new dataset has been built through oversampling, a technique to adjust class distributions and to correct for a bias in the original dataset. This techniques aims to reduce the negative impact of class unbalance on model fitting. The minority classes were randomly sampled (with replacement) in a way to achieve the same size of the majority class (Table 9). The two mental workload indexes (*NASA* and *WP*) and the usability index (*SUS*) were treated as independent variables (features) and they were both employed individually and in combination to induce models aimed at predicting the five classes of objective performance (Fig 13). Four different families of learning classification techniques were adopted to predict the objective performance:

- information-based learning: decision trees (Recursive Partitioning) [100, 101]
  - with the Gini impurity index
  - with the information gain entropy measure
- similarity-based learning: k-Nearest Neighbors (euclidean distance)
- probability-based learning: Naive Bayes
- error-based learning: Support Vector Machine [102, 103].
  - with a radial basis function kernel
  - with a polynomial function kernel

**Fig 14:**
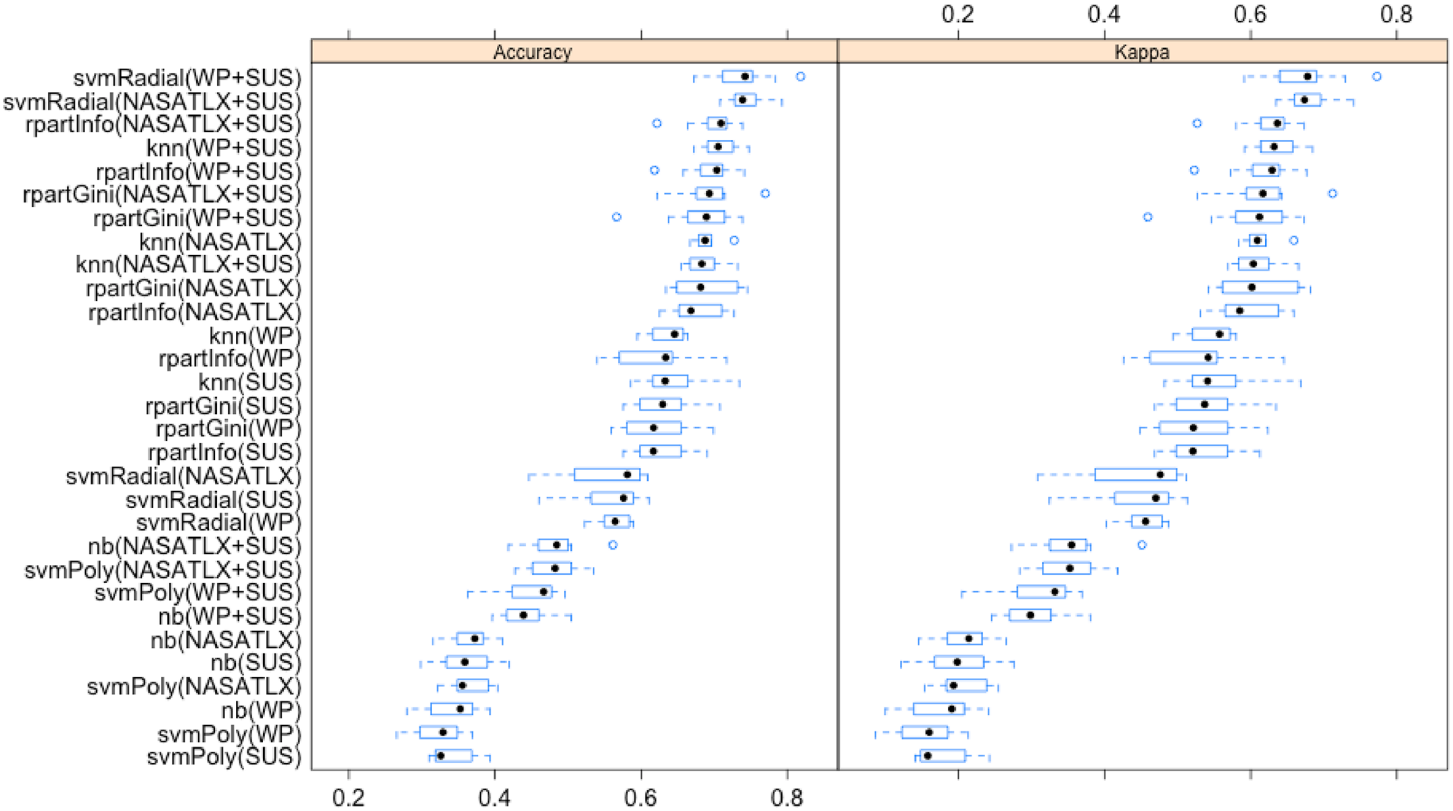
Distribution of the accuracies of individual and combined induced models ordered by mean.

The independent features were normalised with unity-based normalisation (min/max algorithm) in the range [0..1] ∈ ℜ to facilitate the training of models. 10-fold stratified cross validation has been used in the training phase therefore the oversampled dataset was divided in 10 folds and in each fold, the original ratio of the distribution of the objective performance classes (Fig 12, Table 9) was preserved. 9 folds were used for training a model and the remaining fold for testing it against accuracy. This was repeated 10 times shifting the testing fold. Through this approach, 10 models were induced each with an associated classification accuracy. Thus 10 accuracy values were generated for each machine learning technique and for each combination of independent features (Fig 14). Table 10 lists these values, for the individual models (containing only the mental workload or usability feature) against the combined models (containing both the mental workload and the usability features), grouped by classification technique and ordered by mean. Importantly, training sets (a combination of 9 folds) and test sets (the remaining holdout set) were kept the same across the classification techniques and the different combination of independent features (paired 10-fold CV). This was essential to perform a fair comparison of the different trained models using the same data of training and test sets.

**Table 10:** Ordered distributions of accuracies of trained models grouped by learning technique (combined highlighted).

Model	Ind. Features	Min.	1 Q.	Median	Mean	3 Q.	Max.
svmRadial	(NASATLX+SUS)	0.71	0.73	0.74	0.74	0.75	0.79
svmRadial	(WP+SUS)	0.67	0.71	0.74	0.74	0.75	0.82
svmRadial	(WP)	0.52	0.55	0.56	0.56	0.58	0.59
svmRadial	(SUS)	0.46	0.53	0.58	0.56	0.59	0.61
svmRadial	(NASATLX)	0.45	0.52	0.58	0.56	0.60	0.61
knn	(WP+SUS)	0.67	0.69	0.71	0.71	0.72	0.75
knn	(NASATLX)	0.67	0.68	0.69	0.69	0.70	0.73
knn	(NASATLX+SUS)	0.65	0.67	0.68	0.69	0.70	0.73
knn	(SUS)	0.59	0.62	0.63	0.64	0.66	0.73
knn	(WP)	0.59	0.62	0.65	0.64	0.66	0.66
rpartInfo	(NASATLX+SUS)	0.62	0.69	0.71	0.70	0.72	0.74
rpartInfo	(WP+SUS)	0.62	0.69	0.70	0.69	0.71	0.74
rpartInfo	(NASATLX)	0.62	0.65	0.67	0.68	0.71	0.73
rpartInfo	(SUS)	0.58	0.60	0.62	0.62	0.65	0.69
rpartInfo	(WP)	0.54	0.58	0.63	0.62	0.64	0.72
rpartGini	(NASATLX+SUS)	0.62	0.68	0.69	0.69	0.71	0.77
rpartGini	(NASATLX)	0.63	0.65	0.68	0.69	0.73	0.75
rpartGini	(WP+SUS)	0.57	0.66	0.69	0.68	0.71	0.74
rpartGini	(SUS)	0.58	0.60	0.63	0.63	0.65	0.71
rpartGini	(WP)	0.56	0.58	0.62	0.62	0.65	0.70
nb	(NASATLX+SUS)	0.42	0.46	0.48	0.48	0.50	0.56
nb	(WP+SUS)	0.40	0.42	0.44	0.44	0.46	0.50
nb	(NASATLX)	0.32	0.35	0.37	0.37	0.38	0.41
nb	(SUS)	0.30	0.33	0.36	0.36	0.39	0.42
nb	(WP)	0.28	0.31	0.35	0.34	0.37	0.39
svmPoly	(NASATLX+SUS)	0.43	0.45	0.48	0.48	0.50	0.54
svmPoly	(WP+SUS)	0.36	0.43	0.47	0.45	0.48	0.50
svmPoly	(NASATLX)	0.32	0.35	0.36	0.36	0.39	0.40
svmPoly	(SUS)	0.31	0.32	0.33	0.34	0.36	0.39
svmPoly	(WP)	0.27	0.30	0.33	0.32	0.35	0.37

From Table 10, most of the combined models (highlighted in blue), achieved almost always a higher accuracy than the individual models. However, to formally test hypothesis 2, the 10-fold cross-validated paired Wilcoxon statistical test has been chosen for comparing two matched accuracy distributions and to assess whether their population mean ranks differ [104]. This is a paired difference test which is a non-parametric alternative to the paired Student’s t-test. This has been selected because the population of accuracies (obtained testing each holdout set) was assumed to be not normal. Table 11 lists the accuracies achieved by each induced model, ordered by mean and grouped by classification learning technique.

**Table 11:** Wilcoxon test of distributions of accuracies ordered by independent features with 95% confidence intervals (statistically significant different models highlighted).

	Indipendent Features		Accuracy (mean)			
Classifier	Model 1	Model 2	Model 1	Model 2	p-value	Impact
nb	(NASA)	(NASA+SUS)	0.39	0.51	0.0020	yes
knn	(NASA)	(NASA+SUS)	0.70	0.71	0.7263	no
svmRadial	(NASA)	(NASA+SUS)	0.60	0.74	0.0020	yes
svmPoly	(NASA)	(NASA+SUS)	0.36	0.49	0.0059	yes
rpartGini	(NASA)	(NASA+SUS)	0.65	0.68	0.0840	no
rpartInfo	(NASA)	(NASA+SUS)	0.66	0.71	0.0645	no
nb	(WP)	(WP+SUS)	0.34	0.42	0.0039	yes
knn	(WP)	(WP+SUS)	0.66	0.71	0.0526	no
svmRadial	(WP)	(WP+SUS)	0.55	0.71	0.0020	yes
svmPoly	(WP)	(WP+SUS)	0.35	0.47	0.0059	yes
rpartGini	(WP)	(WP+SUS)	0.65	0.64	0.6462	no
rpartInfo	(WP)	(WP+SUS)	0.66	0.64	0.6953	no
nb	(SUS)	(NASA+SUS)	0.36	0.51	0.0039	yes
knn	(SUS)	(NASA+SUS)	0.66	0.71	0.0144	yes
svmRadial	(SUS)	(NASA+SUS)	0.55	0.74	0.0020	yes
svmPoly	(SUS)	(NASA+SUS)	0.33	0.49	0.0020	yes
rpartGini	(SUS)	(NASA+SUS)	0.60	0.68	0.0059	yes
rpartInfo	(SUS)	(NASA+SUS)	0.60	0.71	0.0020	yes
nb	(SUS)	(WP+SUS)	0.36	0.42	0.0129	yes
knn	(SUS)	(WP+SUS)	0.66	0.71	0.0092	yes
svmRadial	(SUS)	(WP+SUS)	0.55	0.71	0.0020	yes
svmPoly	(SUS)	(WP+SUS)	0.33	0.47	0.0020	yes
rpartGini	(SUS)	(WP+SUS)	0.60	0.64	0.0059	yes
rpartInfo	(SUS)	(WP+SUS)	0.60	0.64	0.1934	no

In most of the cases, the combined models always yielded statistically higher classification accuracies than the individual models. In particular, perception of usability alone, as measured by *SUS* was nearly always the worst in predicting objective performance. The addition of a mental workload index to it (either *NASA* or *WP*) significantly enhanced the prediction of objective performance. The experienced mental workload, according to the *NASA* − *TLX* measure was half of the times sufficient to predict objective performance alone. In the other cases, the perceived usability, measured by the *SUS* index, was able to add predictive capacity to the individual models. A similar behaviour occurred with the other measure of mental workload, namely the *WP* index, which was able to predict objective performance individually half of the times. This empirical evidence suggests that indexes of experienced mental workload and perceived usability can be jointly employed to explain objective performance better than when employed individually. In particular, experience mental workload seems to explain larger variance than perception of usability, when both taken into account, as independent variables, to predict classes of objective performance.

### 2.3 Testing hypothesis 3

In order to test hypothesis 3, an investigation of the impact of the attributes used to assess usability (by the *SUS* measure) and the attributes used to assess mental workload (by the *NASA* and the *WP* measures) on the objective performance achieved by user has been conducted. This is a similar experiment as the one conducted for testing hypothesis 2 (section 2.2), but instead of using overall usability and mental workload indexes, as independent features, their dimensions were used. In other words, the questions of Tables A1, A2, A3 in S1 Appendix, were used as independent features. The same classification techniques used in the experiment set for testing hypothesis 2 were employed.

**Fig 15:**
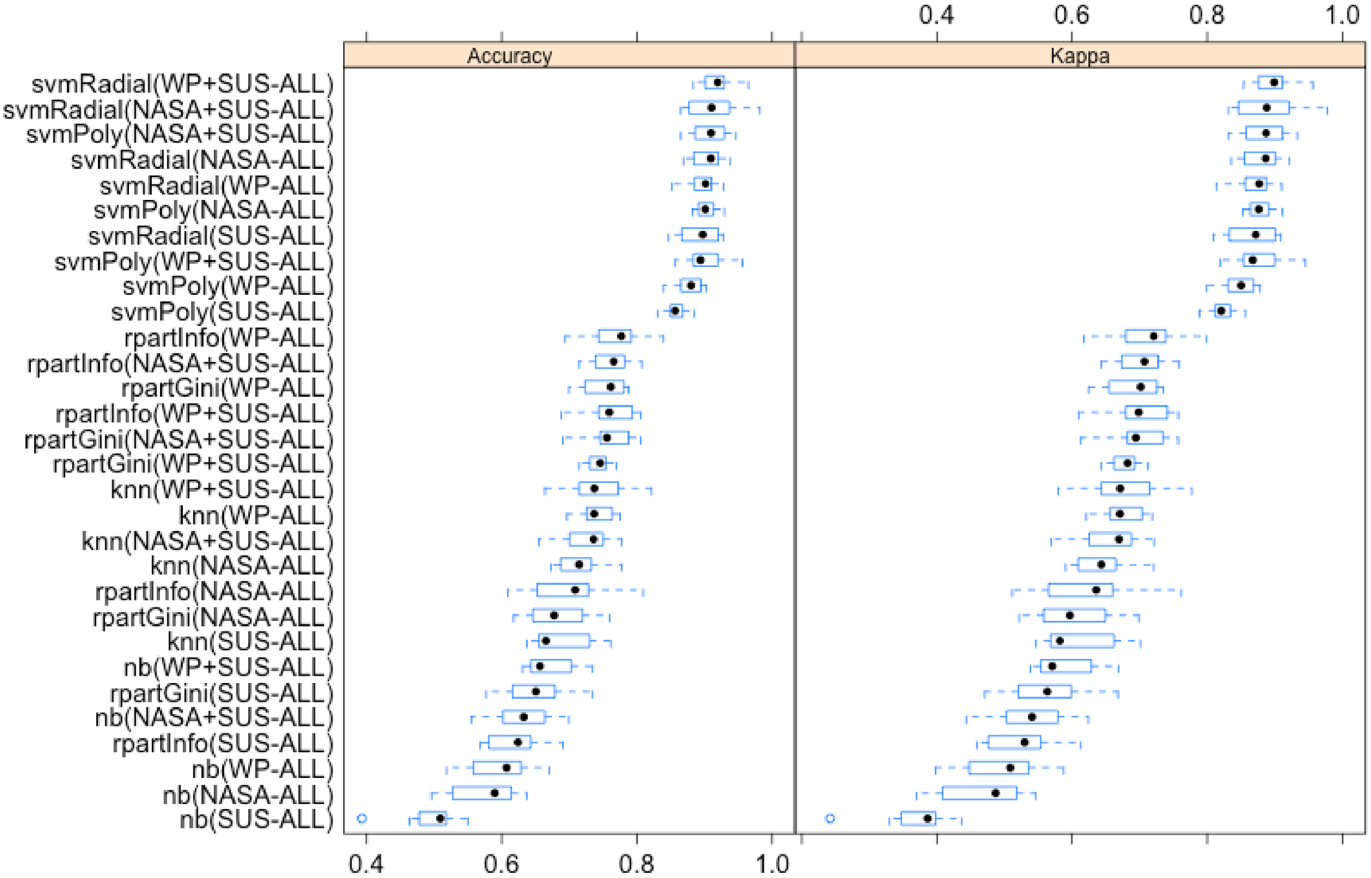
Ordered distributions of accuracies of trained models by mean using full feature sets of original mental workload and usability assessment instruments.

Fig 15 depicts the distributions of the accuracies produced by the models induced by using the selected supervised machine learning classification techniques ordered by means, which are on average higher than the distributions of accuracies obtained in Fig 14. Analytics presented in Table 12 follow the same trend as the findings presented in Table 10 but, on average with higher accuracies. As in the experiment conducted for testing hypothesis 2, the 10-fold cross-validated paired Wilcoxon statistical test has been chosen for comparing two matched accuracy distributions and to assess whether their population mean ranks differ [104]. Table 13 lists these tests comparing models incorporating all the features of the original usability and mental workload instruments, individually and combined. Findings suggest that the incorporation of the factors used within the *SUS* usability assessment technique to the factors used within the *NASA* scale, in a joint model, in most of the cases did not significantly enhance the prediction of objective performance. However, when the factors belonging to *SUS* were added to the factors belonging to *WP* in a joint model, half of the times these did contribute to enhance the prediction of objective performance. Eventually, when mental workload factors, either belonging to the *NASA* or *WP* scales, were added in a joint model to the *SUS* factors, this almost always enhanced the prediction of objective performance.

**Table 12:** Ordered distributions of accuracies of trained models using full feature sets of original mental workload and usability instruments (combined models highlighted).

Model	Independent Features (* = all)	Min.	1 Q.	Median	Mean	3 Q.	Max.
svmRadial	(WP*+SUS*)	0.88	0.90	0.92	0.92	0.93	0.96
svmRadial	(NASA*+SUS*)	0.86	0.89	0.91	0.91	0.94	0.98
svmRadial	(NASA*)	0.87	0.89	0.91	0.91	0.92	0.94
svmRadial	(WP*)	0.85	0.89	0.90	0.89	0.91	0.93
svmRadial	(SUS*)	0.85	0.87	0.90	0.89	0.92	0.93
svmPoly	(NASA*+SUS*)	0.86	0.89	0.91	0.91	0.93	0.95
svmPoly	(NASA*)	0.88	0.89	0.90	0.90	0.91	0.93
svmPoly	(WP*+SUS*)	0.86	0.89	0.89	0.90	0.92	0.96
svmPoly	(WP*)	0.84	0.87	0.88	0.88	0.89	0.90
svmPoly	(SUS*)	0.83	0.85	0.86	0.86	0.87	0.89
rpartInfo	(WP*)	0.69	0.75	0.78	0.77	0.79	0.84
rpartInfo	(NASA*+SUS*)	0.71	0.74	0.77	0.76	0.78	0.81
rpartInfo	(WP*+SUS*)	0.69	0.74	0.76	0.76	0.79	0.81
rpartInfo	(NASA*)	0.61	0.66	0.71	0.70	0.73	0.81
rpartInfo	(SUS*)	0.57	0.59	0.62	0.62	0.64	0.69
rpartGini	(NASA*+SUS*)	0.69	0.75	0.76	0.76	0.78	0.81
rpartGini	(WP*)	0.70	0.73	0.76	0.75	0.78	0.79
rpartGini	(WP*+SUS*)	0.71	0.73	0.75	0.74	0.75	0.77
rpartGini	(NASA*)	0.62	0.65	0.68	0.69	0.71	0.76
rpartGini	(SUS*)	0.58	0.62	0.65	0.65	0.68	0.73
knn	(WP*+SUS*)	0.66	0.71	0.74	0.74	0.77	0.82
knn	(WP*)	0.70	0.73	0.74	0.74	0.76	0.77
knn	(NASA*+SUS*)	0.65	0.70	0.74	0.72	0.75	0.78
knn	(NASA*)	0.67	0.69	0.71	0.71	0.73	0.78
knn	(SUS*)	0.64	0.65	0.67	0.68	0.72	0.76
nb	(WP*+SUS*)	0.63	0.64	0.66	0.67	0.70	0.73
nb	(NASA*+SUS*)	0.55	0.60	0.63	0.63	0.66	0.70
nb	(WP*)	0.52	0.56	0.61	0.60	0.63	0.67
nb	(NASA*)	0.50	0.54	0.59	0.58	0.61	0.64
nb	(SUS*)	0.39	0.48	0.51	0.49	0.52	0.55

**Table 13:** Wilcoxon test of distributions of accuracies ordered by independent features with 95% confidence intervals using mental workload and usability attributes (statistically significant different models highlighted).

	Independent Features (* = all)		Accuracy (mean)			
Classifier	Model 1	Model 2	Model1	Model 2	p-value	Impact
nb	(NASA*)	(NASA*+SUS*)	0.58	0.63	0.0273	yes
knn	(NASA*)	(NASA*+SUS*)	0.72	0.74	0.1934	no
svmRadial	(NASA*)	(NASA*+SUS*)	0.90	0.91	0.7695	no
svmPoly	(NASA*)	(NASA*+SUS*)	0.90	0.90	0.8457	no
rpartGini	(NASA*)	(NASA*+SUS*)	0.71	0.73	0.1309	no
rpartInfo	(NASA*)	(NASA*+SUS*)	0.75	0.74	0.6250	no
nb	(WP*)	(WP*+SUS*)	0.58	0.64	0.0059	yes
knn	(WP*)	(WP*+SUS*)	0.73	0.72	0.3627	no
svmRadial	(WP*)	(WP*+SUS*)	0.89	0.91	0.0273	yes
svmPoly	(WP*)	(WP*+SUS*)	0.87	0.90	0.0225	yes
rpartGini	(WP*)	(WP*+SUS*)	0.71	0.72	0.4316	no
rpartInfo	(WP*)	(WP*+SUS*)	0.74	0.74	0.6101	no
nb	(SUS*)	(NASA*+SUS*)	0.49	0.63	0.0020	yes
knn	(SUS*)	(NASA*+SUS*)	0.69	0.74	0.0137	yes
svmRadial	(SUS*)	(NASA*+SUS*)	0.89	0.91	0.0756	no
svmPoly	(SUS*)	(NASA*+SUS*)	0.85	0.90	0.0059	yes
rpartGini	(SUS*)	(NASA*+SUS*)	0.65	0.73	0.0020	yes
rpartInfo	(SUS*)	(NASA*+SUS*)	0.67	0.74	0.0020	yes
nb	(SUS*)	(WP*+SUS*)	0.49	0.64	0.0020	yes
knn	(SUS*)	(WP*+SUS*)	0.69	0.72	0.0225	yes
svmRadial	(SUS*)	(WP*+SUS*)	0.89	0.91	0.0129	yes
svmPoly	(SUS*)	(WP*+SUS*)	0.85	0.90	0.0092	yes
rpartGini	(SUS*)	(WP*+SUS*)	0.65	0.72	0.0020	yes
rpartInfo	(SUS*)	(WP*+SUS*)	0.67	0.74	0.0195	yes

### 2.4 Internal reliability of measurement scales

**Fig 16:**
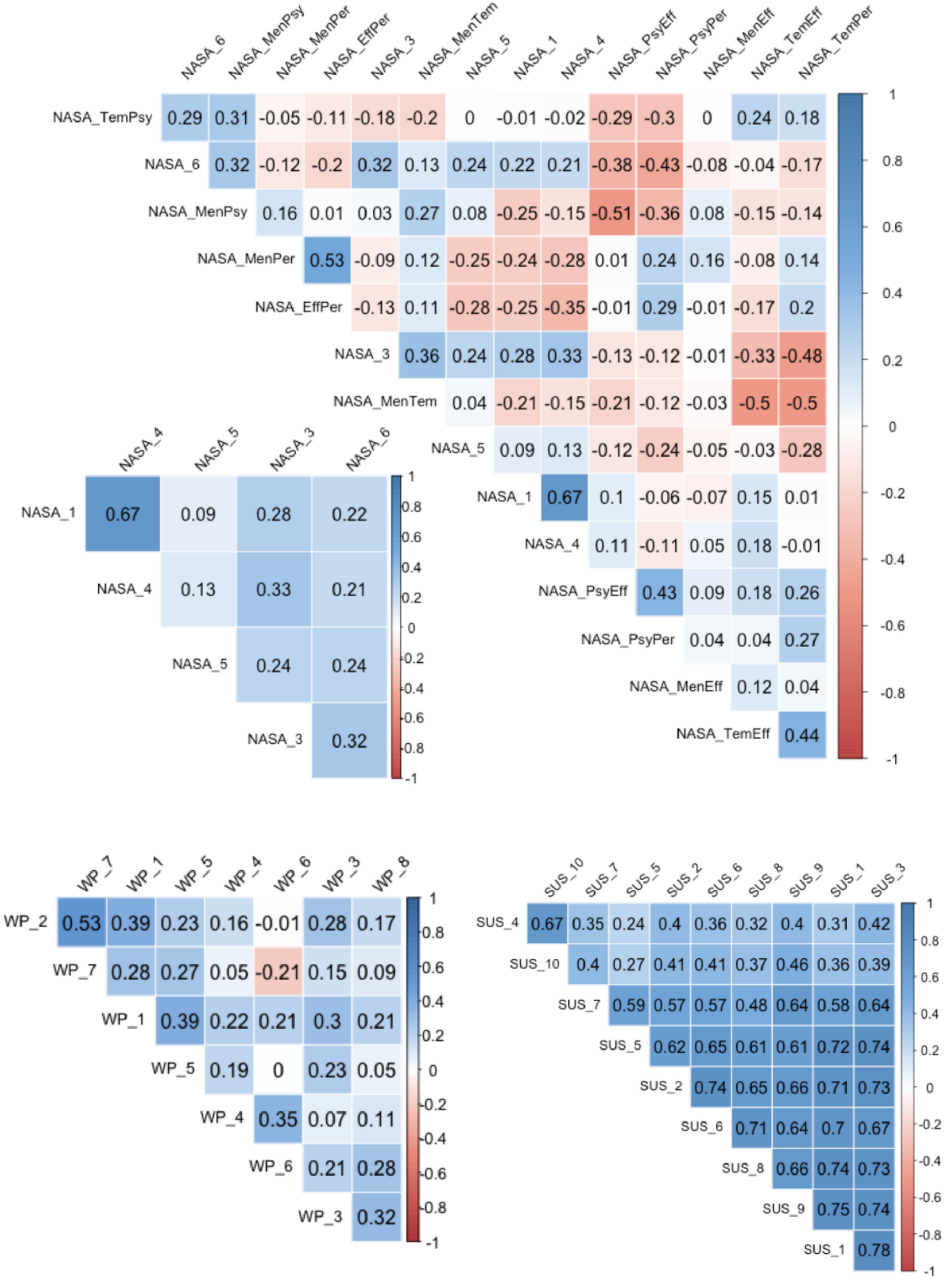
Intra-correlations of mental workload and usability questionnaire items.

In order to enhance the reliability of the findings obtained in this empirical research, the Cronbach’s alpha measure was computed to test the internal consistency of the psychometric instruments used, namely the System Usability Scale (*SUS*) and the mental workload assessment instruments, namely the Nasa Task Load Index (*NASA* − *TLX*) and the Workload Profile scale (*WP*). The Cronbach’s alpha is aimed at assessing how well these scales consistently measures what they are supposed to measure. Fig 16 depicts the inter-item correlations of the above scales, while Table 14 lists the Cronbach’s Alpha coefficient for each scale.

**Table 14:** Cronbach’s Alpha of the mental workload and usability questionnaire items.

NASA	NASA+pairwise	WP	SUS
0.65	0.54	0.64	0.93

Clearly, the System Usability Scale is the most reliable, with a Cronbach’s Alpha coefficient of 0.93, in line with findings present in the literature [33, 105]. The Nasa Task Load index follows, with a lower reliability of 0.65 (using only the close ended questions) and 0.54 (using also the binary pair-wise comparisons) that indicates a questionable internal consistency of the scale. However, this is also in line with studies in the literature [106]. An important point is that the *NASA* − *TLX* scale as predominantly used in the field of transportation and safety critical systems [9] with little application in Human-Computer Interaction and within the arena of modern daily digital systems. The reliability of the Workload Profile (0.64) was aligned to the reliability of the *NASA* − *TLX* and these two scales had a fair concurrent validity (Correlation of 0.55 from Table 14).

## 3 Discussion

The results obtained in the previous sections are summarised and they are aligned to the research hypotheses previously set in Tables 2 and 3. Findings are critically discussed, including information about their statistical significance. Eventually, their implication to the broader field of Human-Computer Interaction is described.

•*H*_1_: *Usability and Mental workload are two uncorrelated constructs capturing difference variance (measured by self-reporting techniques—SUS,* *NASA-TLX, WP)*.

This has been tested by an in depth correlation analysis, both parametric and nonparametric, which confirmed that the two constructs are not correlated. The obtained correlation coefficients of Table 7 suggest that there is no linear correlation between the perception of usability and the experienced mental workload by users, with Pearson coefficients close to zero. Similarly, data suggests there is no tendency for usability to either monotonically increase or decrease when mental workload increases, with Spearman coefficients close to zero. The significance levels obtained for these correlations confirm that *SUS* and the *NASA* are significantly uncorrelated, fully accepting hypothesis *H*_1_(*a*). However, the absent correlation obtained between *SUS* and *WP* cannot be confirmed by statistical significance, thus the acceptance of sub-hypothesis *H*_1_(*b*) is with reservation. The correlation analysis has been strengthened by computing sub-correlations of data respectively by experimental task, users and objective performance class (Tables 6–8). Once more, no consistent pattern on the relationship between perception of usability and mental workload emerged, enforcing the belief that the two constructs are uncorrelated and capture difference variance.

•*H*_2_: *A unified model incorporating a usability and a MWL measure can significantly enhance the accuracy of the prediction of objective performance than the individual usability and MWL models.*

This has been tested by inducing unified and individual models, using four supervised machine learning classification techniques, to predict the objective performance achieved by users (five classes of performance of Table 1). These models were trained with 10-fold cross validation, a well known model validation approach aimed at assessing how the results of a statistical analysis will generalise to an independent data set. For each classification technique, 10 accuracies were computed forming a distribution. Distributions of accuracies were subsequently compared using the 10-fold cross-validated paired Wilcoxon statistical test to assess whether their population mean ranks differ, with a significance level of 0.05. The unified models, including the measure of usability and one measure of mental workload, as independent features, were most of the times able to predict objective user performance, as dependent feature, statistically significantly better than the individual models (containing only one independent features, either usability or MWL). In details, on one hand, when the *NASA* measure was added to the *SUS* measure, it was always effective in significantly enhance the accuracy of the predictions, therefore sub-hypotheses *H*_2_(*a*, *b*) can be fully accepted. On the other hand, when *SUS* was added to a measure of mental workload, it enhanced the prediction of objective performance only half of the times. As a consequence, sub-hypotheses *H*_2_(*c*, *d*) cannot be fully accepted. This suggests that the NASA-TLX, as a measure of mental workload, has a higher capacity to explain objective performance than SUS, a measure of usability.

•*H*_3_: *A hybrid model incorporating features of a usability measure and features of a mental workload measure can significantly enhance the prediction of objective performance than models incorporating only usability or mental workload features.*

This has been tested by inducing models containing the features of the System Usability Scale and the features of a measure of mental workload (either *NASA* or *WP*), firstly individually and then combined (hybrid model). The same four supervised machine learning classification techniques have been employed to train models to predict the objective performance achieved by users (five classes of performance of Table 1). These models were trained again with 10-fold cross validation in order to assess how the results will generalise to independent data. For each classification technique, 10 accuracies were computed forming a distribution. Distributions of accuracies were again compared using the 10-fold cross-validated paired Wilcoxon statistical test to assess whether their population mean ranks differ, with a significance level of 0.05. The hybrid models achieved, half of the times, a significantly higher classification accuracy than the individual models. In details, features of mental workload, when added to features of usability, nearly always statistically significantly enhanced the prediction of objective performance, therefore in favour of the acceptance of sub-hypotheses *H*_3_(*a*, *b*). However, on one hand, when features of mental workload belonging to the *WP* measure were added to features belonging to the *SUS* measure, only half of the times these significantly increased the classification of objective performance. On the other hand, when features of mental workload belonging to the *NASA* measure were added to features belonging to the *SUS* measure, nearly always did not enhance the prediction of objective performance. These results suggest that sub-hypothesis *H*_3_(*d*) cannot be fully accepted and sub-hypothesis *H*_3_(*c*) has to be rejected. It turns out that the features used within the NASA Task Load Index measure are powerful in predicting objective performance alone strengthening the fact that usability and mental workload measure two different aspects of user experience.

Table 15 summarises the acceptance status of the sub-hypotheses, whether they can be accepted, rejected or are subject to uncertainty.

**Table 15:** Formal description of research hypotheses and their acceptance status (*corr* a correlation coefficient and *acc* the accuracy of the model’s prediction).

	formal description	status
*H*_1_	a) *corr*(*SUS*, *NASATLX*) = 0	✓
	b) *corr*(*SUS*, *WP*) = 0	✓
*H*_2_	a) *acc*(*SUS* → *OP*) < *acc*(*SUS*, *NASATLX* → *OP*)	✓
	b) *acc*(*SUS* → *OP*) < *acc*(*SUS*, *WP* → *OP*)	✓
	c) *acc*(*NASATLX* → *OP*) < *acc*(*SUS*, *NASATLX* → *OP*)	?
	d) *acc*(*WP* → *OP*) < *acc*(*SUS*, *WP* → *OP*)	?
*H*_3_	a) *acc*(*SUS*_1,..,10_ → *OP*) < *acc*(*SUS*_1,..,10_, *NASA*_1,..,6_ → *OP*)	✓
	b) *acc*(*SUS*_1,..,10_ → *OP*) < *acc*(*SUS*_1,..,10_, *WP*_1,..,8_ → *OP*)	✓
	c) *acc*(*NASA*_1,..,6_ → *OP*) < *acc*(*SUS*_1,..,10_, *NASA*_1,..,6_ → *OP*)	X
	d) *acc*(*WP*_1,..,8_ → *OP*) < *acc*(*SUS*_1,..,10_, *WP*_1,..,8_ → *OP*)	?

In summary, empirical evidence from this study suggests that there is no relationship between the perception of usability and the mental workload experienced by users on a set of web-based tasks executed on selected interfaces. Findings suggests that the two constructs seem to describe two not overlapping phenomena, sharing very little variance. The implication of this is that perception of usability and levels of experienced mental workload could be jointly employed to enhance the description of user experience. This is particularly relevant, for instance, in those scenarios in which humans, interacting with technologies, are people with cognitive disabilities or elderly, or when a graphical interface is presented on a desktop screen or a mobile device. Here, perception of usability can be high, but underlying tasks might impose non optimal levels of mental workload on users. Similarly, users can experience optimal mental load while executing underlying tasks, but might not perceive underlying interactive systems usable. In turn, the consideration of usability and mental workload as two distinct constructs can aid designers to build interactive technologies better aligned to the human mental limited capacities and that can maximise human performance. For example, during design phases, a designer can perform A/B testing of an interactive system by assessing mental workload and usability. In turn, this will generate a richer spectrum of feedback that can be taken into account to improve system design and optimise the performance of its users. The contributions of this research are to offer a new perspective on the application of mental workload to traditional usability inspection methods, and a richer approach to explain the human-system interaction and support its design.

## 4 Conclusion

This study attempted to investigate the correlation between the perception of usability and the mental workload imposed by typical tasks executed over three popular web-sites: Youtube, Wikipedia and Google. A literature review on prominent definitions of usability and mental workload was presented, with a particular focus on the latter construct. A well known subjective instrument for assessing usability —the System Usability Scale —and two subjective mental workload assessment procedures —the NASA Task Load Index, and the Workload Profile —have been employed in a primary research study involving 46 subjects. The perception of the usability of the interfaces these subjected interacted upon and the mental workload they have experienced while executing a selection of tasks, over selected interfaces, does not seem to correlate. The obtained empirical evidence strongly supports that usability and mental workload are two non overlapping constructs. Findings suggest that these two constructs can be jointly employed to improve the prediction of human performance, thus enhancing the description of user experience. The implications to the broader field of Human-Computer Interaction include the provision of mental workload as an important concept relevant for the design of interactive technologies better aligned to the human mental limited capacities and that can maximise human performance.

Future work will be devoted to the replication of this primary research on different interfaces and interactive systems. Experiment will be conducted by considering a wider selection of cognitive tasks in terms of temporal length, context (controlled and real-world tasks), complexity and mental resources demand. Similarly, a wider range of subjects is planned, including people affected by motor or cognitive impairments as well elderlies. Eventually, a new hybrid construct for explaining human performance over interactive technologies is envisaged. This new construct will incorporate factors concerned usability as well as human mental workload into a novel unified metric expected to have a higher validity, sensitivity and precision than current ad-hoc measures of user experience.

## Supporting information

S1 Appendix (TEX)
